# A New Perspective on Adsorbent Materials Based Impregnated MgSiO_3_ with Crown Ethers for Palladium Recovery

**DOI:** 10.3390/ijms221910718

**Published:** 2021-10-03

**Authors:** Mihaela Ciopec, Oana Grad, Adina Negrea, Narcis Duteanu, Petru Negrea, Cristina Paul, Catalin Ianăși, Giannin Mosoarca, Cosmin Vancea

**Affiliations:** 1 Faculty of Industrial Chemistry and Environmental Engineering, Politehnica University Timisoara, Bd. V. Parvan, No. 6, 300223 Timisoara, Romania; mihaela.ciopec@upt.ro (M.C.); oana.grad@upt.ro (O.G.); adina.negrea@upt.ro (A.N.); narcis.duteanu@upt.ro (N.D.); petru.negrea@upt.ro (P.N.); cristina.paul@upt.ro (C.P.); 2 Research Institute for Renewable Energy of the Politehnica University Timisoara, Musicescu Street, No. 138, 300774 Timisoara, Romania; 3 “Coriolan Drăgulescu” Institute of Chemistry, Romanian Academy, Bv. Mihai Viteazul, No. 24, 300223 Timisoara, Romania; ianasic@acad-icht.tm.edu.ro

**Keywords:** magnesium silicate, dibenzo18-crown-6, dibenzo 30-crown-10, palladium recovery, adsorption

## Abstract

The study of new useful, efficient and selective structures for the palladium ions’ recovery has led to the development of a new series of macromolecules. Thus, this study presents a comparative behavior of two crown benzene ethers that modify the magnesium silicate surface used as adsorbent for palladium. These crown ethers are dibenzo18-crown-6 (DB18C6) and dibenzo 30-crown-10 (DB30C10). The obtained materials were characterized by scanning electron microscope (SEM), energy-dispersive X-ray spectroscopy (EDX) and Fourier-transform infrared spectroscopy (FT-IR). The specific surface area (BET) and point of zero charge (PZC) of the two materials were determined. The palladium ions’ recovery from synthetic aqueous solutions studies aimed to establish the adsorption mechanism. For this desideratum, the kinetic, equilibrium and thermodynamic studies show that MgSiO_3_-DB30C10 have a higher adsorption capacity (35.68 mg g^−1^) compared to MgSiO_3_-DB18C6 (21.65 mg g^−1^). Thermodynamic studies highlight that the adsorption of Pd(II) on the two studied materials are spontaneous and endothermic processes. The positive values of the entropy (ΔS^0^) suggest that the studied adsorption processes show a higher disorder at the liquid/solid interface. Desorption studies were also performed, and it was found that the degree of desorption was 98.3%.

## 1. Introduction

The ability of the crown ethers to form complexes with certain metal ions is well recognized [1,2]. “Crown ether” is a generic name given to macrocyclic polyethers that contain ethylene bridges separating electronegative oxygen ions. When the metal cation enters the crown ether cavity or gets stuck between two crown ether molecules it becomes a lipophilic species. This property of crowns makes them very effective for removing cations [3].

The removal or recovery of metal ions from aqueous solutions is a topic of great interest due to its high impact on the environment [4]. One such example of high importance is the elimination of toxic heavy metals such as Cd(II), Hg(II) Pb(II) [5] or toxic non-metals such as As(III) or As(V) from wastewater or even from natural waters [6]. In addition, an increased interest is given to efficient recovery alternatives of the precious metals used in the industry such as Pt(IV), Au(III) or Pd(II) [7]. The Pd(II) recovery mechanism by adsorption from aqueous solutions in general and from dilute solutions in particular depends on the nature of the adsorbent material. In order to obtain good yields of the adsorption properties it is advisable that the adsorbent material provides a high selectivity, taking into account the relatively large number of naturally occurring rare-earth elements, and the importance of their recovery. The obtained results, showing a large number and diversity of existing materials, were greeted with enthusiasm. Among these, are biological materials, oxides, activated carbon, waste and by-products of industrial, commercial or natural polymeric resins, etc.

There are multiple methods for recovering/removing metal cations: crystallization, precipitation, ion exchange, solvent extraction, reduction or even molten salt distillation [8,9].

An interesting approach on metal cation recovery is the use of complexing agents that can be easily separated from the solution using adsorption and complexation processes. In recent years, macrocyclic crown ethers have been widely used as extractants, having promising efficiency due to their complexing abilities [10,11,12,13]. This paper proposes the study of two such crowns ethers, namely dibenzo-18-crown-6 (DB18C6) and dibenzo-30-crown-10 (DB30C10).

Magnesium silicate is known to be used as a support functionalized with various active groups such as −NH_2_, −SH, −PO_3_H_2_, etc., in order to obtain high-performance materials for the metal ions’ recovery/removal from aqueous solutions [14,15].

Palladium is known as a platinum group metal, very important in the production process of many electronic components such as diodes, transistors, integrated circuits, memories and semiconductors, but also in the production of multilayer ceramic capacitors, alloys for electrical contacts, electrical relays, systems switching, telecommunication devices, etc. [16,17]. Palladium is also part of dental amalgams (gold-silver-copper-palladium) [18], and can be used as a catalyst in a number of industrial processes [16,17], in the manufacturing of jewelry and coins [19] and even in the pharmaceutical field [20]. As a result of these various usages and of the fact that the resources of palladium are declining, the recovery of this important metallic cation is mandatory.

This paper proposes two new materials designed for palladium recovery from waste solutions, knowing the economic value of this noble metal. These new adsorbents are obtained by crown ethers (DB18C6 and DB30C10) grafting on the MgSiO_3_ surface. The study explores a comparison between these new materials and also examines other adsorbents mentioned in the literature for palladium recovery, highlighting their higher adsorption capacity.

## 2. Results and Discussion

### 2.1. Characterization of MgSiO_3_ Modified with Crown Ethers

#### 2.1.1. Scanning Electron Microscopy (SEM)

**Figure 1:**
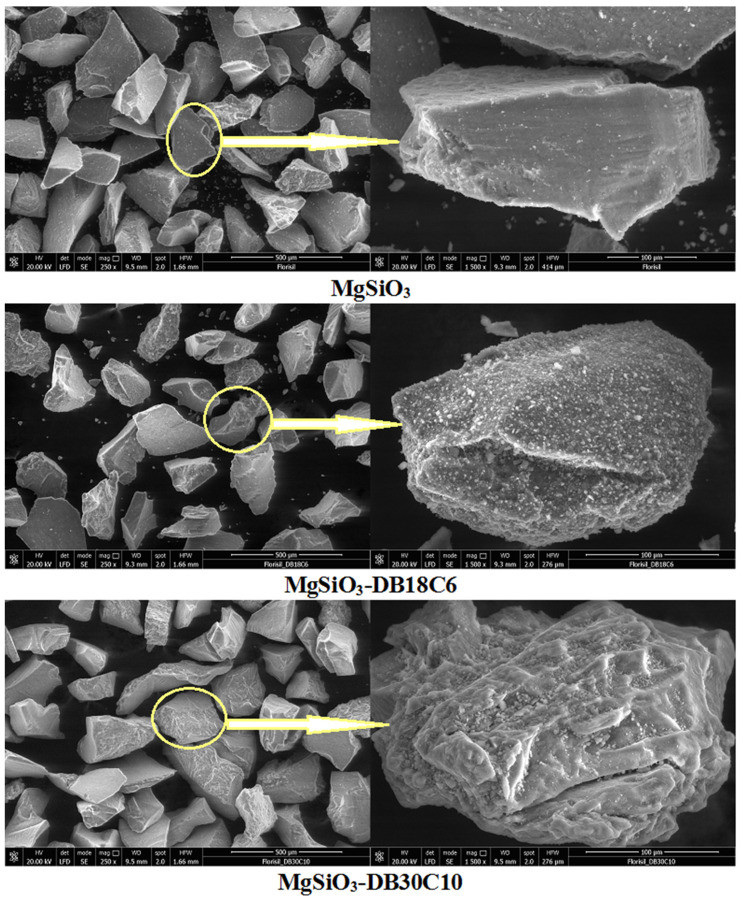
SEM images of the adsorbent before and after functionalization.

SEM images of the inorganic support MgSiO_3_ before and after functionalization with the two crown ethers are shown in Figure 1 at two different magnifications: ×250 and ×1500.

The SEM image of MgSiO_3_ shows an initially porous and homogeneous surface [21,22], that changes after the functionalization by impregnation with the crown ether, as can be observed in the high magnification images.

Comparing the SEM images before and after functionalization, the small white spots (microgranules) can be observed, which can be attributed to the presence of crown ethers on the surface of the support material used. DB30C10 is better dispersed on the adsorbent surface, showing a more uniform distribution compared to the DB18C6 crown ether [14,15].

The MgSiO_3_-DB18C6 surface is much more porous compared to the MgSiO_3_-DB30C10 surface, which becomes glossier and denser. This finding can be attributed to the fact that the impregnation of MgSiO_3_ with DB18C6 is done in points forming an uneven surface, while the DB30C10 coating of the substrate surface is more uniform.

#### 2.1.2. Energy-Dispersive X-ray Spectroscopy (EDX)

**Figure 2:**
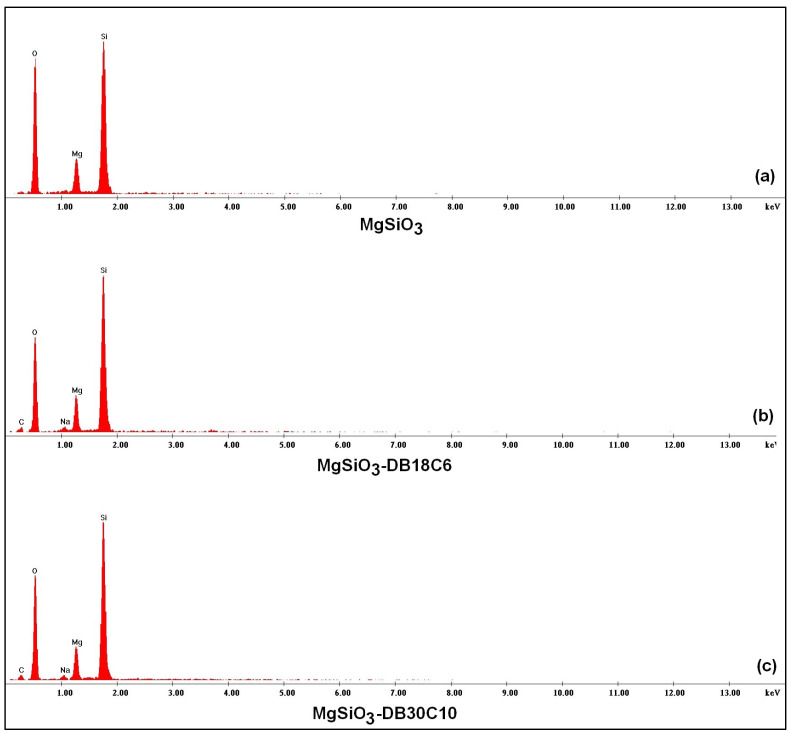
EDX spectroscopy of the adsorbent before and after functionalization: (**a**) MgSiO_3_, (**b**) MgSiO_3_-DB18C6 and (**c**) MgSiO_3_-DB30C10.

The EDX spectra (Figure 2) confirm the functionalization of the MgSiO_3_ support with the two crowns. Figure 2a illustrates the specific peaks of the adsorbent (Mg, Si and O) in the range 2–4 keV. The peak C (also visible in Figure 2a) comes from carbon in self-adhesive carbon paper, and in the case of EDX spectra in Figure 2b,c, additionally from crown ethers.

#### 2.1.3. Fourier-Transform Infrared Spectroscopy (FT-IR)

**Figure 3:**
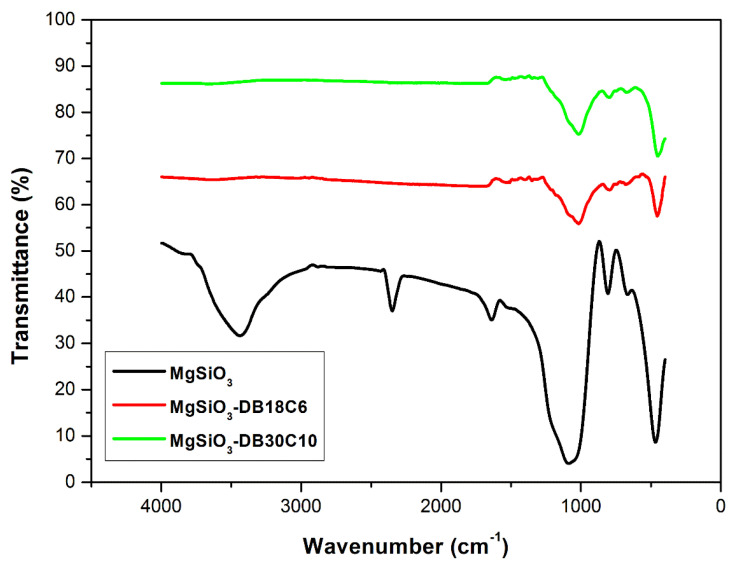
FT-IR spectra of MgSiO_3_, MgSiO_3_-DB18C6 and MgSiO_3_-DB30C10.

A specific method used to confirm the presence of the crown ethers on the surface of the support material is the Fourier transform infrared spectroscopy (FT-IR). The FT-IR infrared spectra of the uncoated adsorbent and both functionalized materials were recorded (Figure 3), in the wavenumbers range 4000 to 400 cm^−1^, at a resolution of 2 cm^−1^ and 40 scans, using KBr pills.

The FT-IR spectrum of MgSiO_3_ (florisil) shows the specific bands attributed to the following: the presence of the O-H stretching frequency of the surface silanol group and also to adsorbed water molecules at ~3400 cm^−1^, the angular vibration of the water molecule at 1650 cm^−1^, the siloxane stretching frequency, ν(Si–O–Si), at 1100 cm^−1^ and the Si–OH bending frequency band at 800 cm^−1^ [23].

The FT-IR spectra of the functionalized materials show an attenuation of the specific MgSiO_3_ vibrations after functionalization. The vibrations of the crown ethers are of low intensity due to the relatively small amount of crown ethers used (MgSiO_3_: crown ethers ratio: 10:1). Thus, at 1600–1500 cm^−1^ there is a small specific vibration attributed to the C–H bond in the benzene ring from the crown ethers [24]. The small vibrations recorded at 1250–1140 cm^−1^ are specific to the C–O–C and C_6_H_5_–O–C bond in the crown ethers [25].

#### 2.1.4. Brunauer–Emmett–Teller (BET) Surface Area Analysis

**Figure 4:**
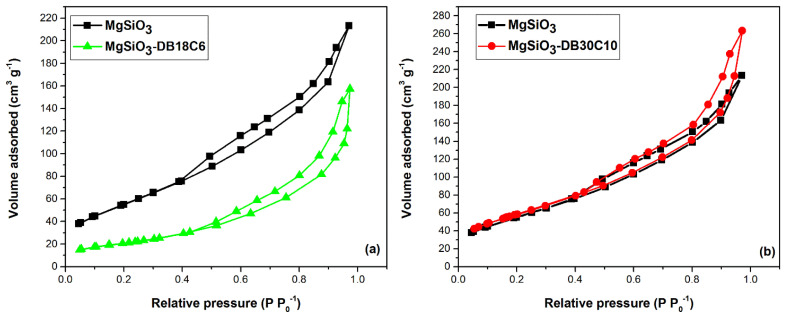
The N_2_ adsorption–desorption isotherms: (**a**) MgSiO_3_ and MgSiO_3_-DB18C6, (**b**) MgSiO_3_ and MgSiO_3_-DB30C10.

A comparison between the N_2_ adsorption–desorption isotherms of MgSiO_3_ and MgSiO_3_-DB18C6 and MgSiO_3_ and MgSiO_3_-DB16C6, respectively, is presented in Figure 4.

Table 1 shows the calculated parameters specific to the adsorption isotherms.

Evaluating the data obtained and comparing with IUPAC [26] confirms that all materials are specific to mesoporous adsorbents generating a type IVa isotherms. The hysteresis obtained for this type of material is usually encountered when the pore width exceeds a certain critical width.

By analyzing the textural parameters with the NLDFT model, we observe that the pore size distribution indicates only mesoporous in the case of pure MgSiO_3_. When DB18C6 and DB3010 are added, a decrease in size from 3.87 nm (for pure MgSiO_3_) to 1.69 nm (DB18C6) to 2.89 nm (DB30C10) can be observed.

The surface area determined using the BET method indicates a value of 205 (m^2^ g^−1^) for pure MgSiO_3_. When DB30C10 is added, the surface area shows a slight increase to 215 (m^2^ g^−1^) suggesting that a few new pores are formed. In the case of a sample functionalized using DB18C6, the surface area decreases three times from the pure MgSiO_3_ obtaining a value of 75 (m^2^ g^−1^).

From the last point of isotherms, the total pore volume is calculated. The initial sample MgSiO_3_ shows a value of 0.33 (cm^3^ g^−1^) with pores smaller than 66.6 nm. When DB18C6 is added we obtain a higher value of 0.03 (cm^3^ g^−1^) with pores smaller than 2.65 nm. In the case of sample DB30C10, we observe a clear decrease of total pore volume with a value of 0.48 (cm^3^ g^−1^) and pores smaller than 69.5 nm indicating that all pores are almost filled.

The fact that if MgSiO_3_ is functionalized with DB18C6 it leads to a decrease of pore size, specific surface area as well as pore volume, suggests that this crown can block the pores of the support. The functionalization with DB30C10 increases the pore size, suggesting that these large crown ether molecules cannot penetrate the pores and stick to the surface of the support.

#### 2.1.5. Point of Zero Charge (PZC)

It is a known fact that for materials with adsorbent properties, the knowledge of their acid-base properties is an important factor in establishing their use. The point of zero charge, pH_PZC_, is the point at which the surface concentration of the negatively charged groups is equal to the surface concentration of the positively charged groups.

**Figure 5:**
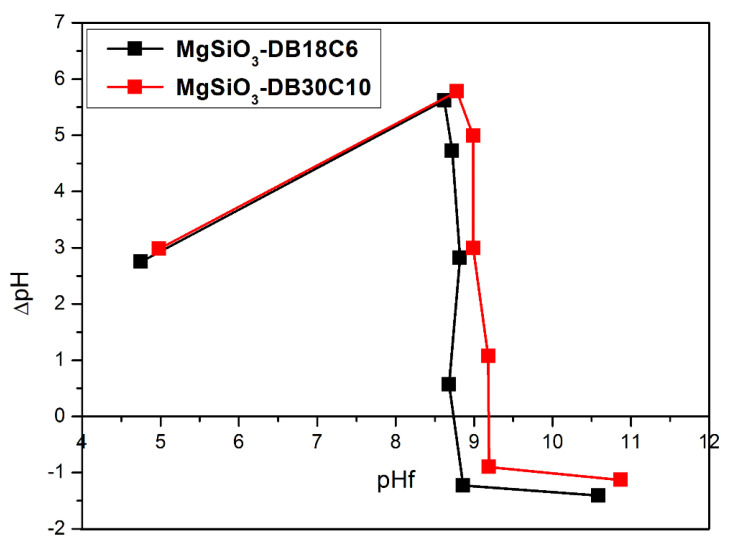
Point of zero charge (PZC) for the functionalized adsorbent.

The pH_PZC_ value associated with each functionalized support was calculated from the plot of ΔpH (pH_f_–pH_i_) against the final pH value (pH_f_) (Figure 5).

The pH_PZC_ of the MgSiO_3_-DB18C6 was found to be 8.7 and pH PZC of MgSiO_3_-DB30C10 was found to be 9.2. For this pH value, both cationic and anionic species can be adsorbed on the surface of the functionalized material. For pH values below the pH_PZC_ value, the surface of the material will be positively charged due to the adsorbed protons, favoring the adsorption of anionic species. For pH values above the pH_PZC_ value, the surface of the material will be negatively charged due to the adsorbed hydroxyl ions, favoring the adsorption of cationic species.

### 2.2. Adsorption Study

With adsorption being an advanced purification method with high efficiency for Pd(II) removal, the experimental studies focus on the development of new materials having advanced adsorbent properties.

The adsorption studies were performed in the static regime, establishing the following characteristic parameters: pH, contact time, temperature, adsorption capacity, maximum adsorption concentration as well as the behavior of materials in the desorption process.

#### 2.2.1. pH Effect

**Figure 6:**
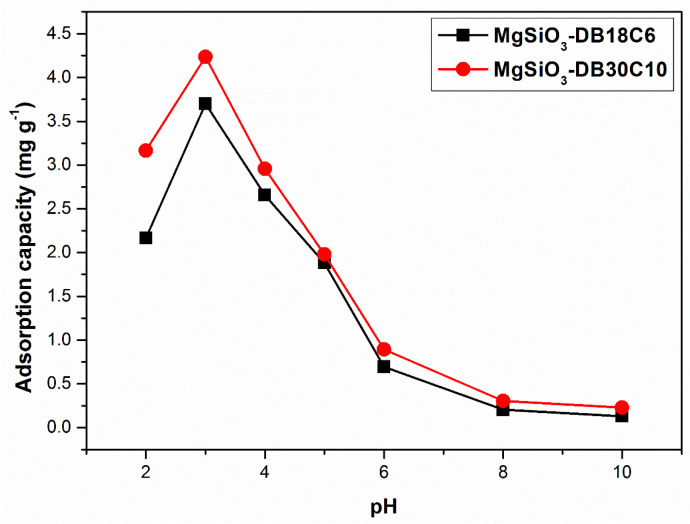
pH effect upon the adsorption capacity of the functionalized adsorbents.

In adsorption studies it is important to know the predominant species at a certain pH value. Depending on the pH, these species are either predominant or not in the aqueous environment. Figure 6 shows the variation of the adsorption capacity as a function of pH.

For both studied materials, the optimal value for the pH is 3, leading to a maximum adsorption capacity of 4.2 (mg g^−1^) in the case of MgSiO_3_-DB30C10 and 3.7 (mg g^−1^) in the case of MgSiO_3_-DB18C6. As the pH increases, the hydrolysis of Pd^2+^ ions take place according to the reaction:

q Pd^2+^ + p H_2_O ↔ Pd_q_(OH)_p_^(2q-p)^ + p H^+^

with the formation of palladium hydroxide and hydroxo-complexes [27,28].

The decrease of the palladium adsorption capacity at a pH higher than 3 can be a consequence of the formation of a soluble form of palladium hydroxide and, at high concentrations, to precipitation of the palladium hydroxide phase. According to the distribution diagram of the species Pd(II) at pH = 3 the adsorbed species is [Pd(H_2_O)_4_]^2+^ [29].

#### 2.2.2. Contact Time and Temperature Effect

In order to establish the kinetics of the Pd(II) recovery process by adsorption on the two studied materials, MgSiO_3_-DB18C6 and MgSiO_3_-DB30C10, it is very important to know the influence of the contact time on the adsorption capacity. In addition, for the thermodynamics aspects of the adsorption process, it is important to know the effect of temperature on the adsorption capacity.

**Figure 7:**
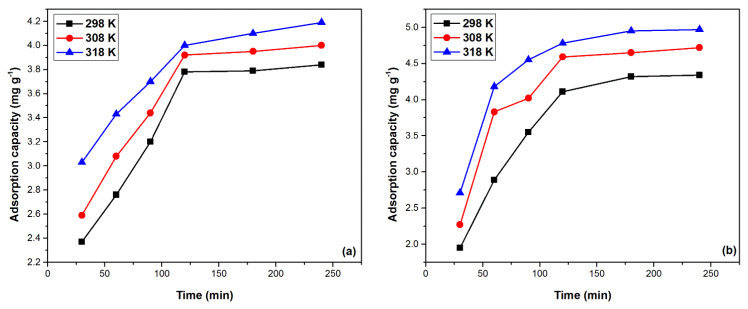
Contact time and temperature effect on the adsorption capacity of the functionalized adsorbents: (**a**) MgSiO_3_-DB18C6, (**b**) MgSiO_3_-DB30C10.

Figure 7 illustrates the effect of contact time and temperature on the adsorption process. As the contact time between adsorbent and adsorbed increases, the adsorption capacity for both materials increase up to a certain point. Thus, after 120 min, the adsorption capacity remains approximately constant, namely for MgSiO_3_-DB18C6 in the range of 3.8–4.0 (mg g^−1^) and for MgSiO_3_-DB30C10 in the range of 4.0–4.8 (mg g^−1^), depending on the temperature. The adsorption process is positively influenced by the temperature. As the temperature increases, in the range of 298–318 K, the adsorption capacity increases, but not significantly, so that studies can be performed at a temperature of 298 K.

#### 2.2.3. Initial Concentration Effect

**Figure 8:**
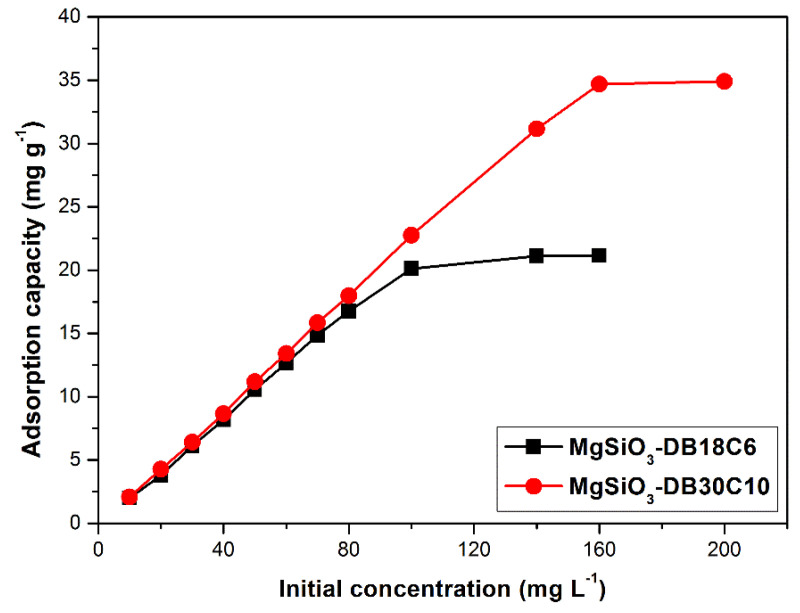
Initial concentration effect on the adsorption capacity of the functionalized adsorbents.

For the initial Pd(II) concentration where equilibrium is reached, adsorption studies are performed for solutions having initial concentrations ranging from 10 to 200 (mg L^−1^), at a temperature of 298 K, for 120 min, pH = 3, using 0.1 g of adsorbent material. The results are presented in Figure 8.

Experimental data show that for the MgSiO_3_-DB18C6 adsorbent, an initial concentration of ~100 (mg L^−1^) leads to a maximum adsorption capacity of ~20 (mg g^−1^) and respectively, for the MgSiO_3_-DB30C10 material, an initial concentration of 160 (mg L^−1^), leads to a maximum adsorption capacity of 34.7 (mg g^−1^). The difference between the two adsorbents’ behavior is generated by the significant size difference between them: the crown ether DB30C10 is larger (almost double the number of O atoms) than DB18C6.

#### 2.2.4. Adsorption Kinetics Study

**Figure 9:**
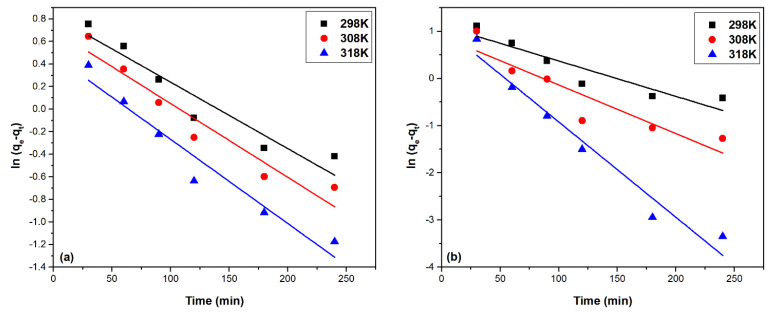
Pseudo-first-order kinetic models: (**a**) MgSiO_3_-DB18C6, (**b**) MgSiO_3_-DB30C10.

**Figure 10:**
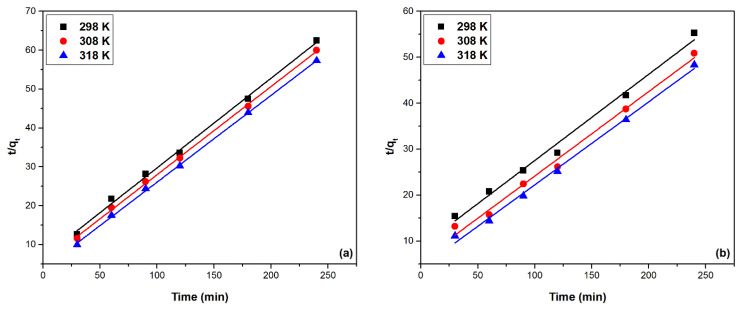
Pseudo-second-order kinetic models: (**a**) MgSiO_3_-DB18C6, (**b**) MgSiO_3_-DB30C10.

The kinetics of the Pd(II) adsorption process, and also the kinetic mechanism governing the Pd(II) adsorption process on the studied adsorbent materials, MgSiO_3_-DB18C6 and MgSiO_3_-DB30C10, is studied using two different kinetic models, namely: pseudo-first-order kinetic model (Lagergren model) and pseudo-second-order kinetic model (Ho-McKay model), respectively [30]. Mathematical modeling of the experimental data is presented in Figure 9 and Figure 10.

Based on the regression coefficient values away from the value 1, being between 0.91 and 0.95 for the material MgSiO_3_-DB18C6 and between 0.84 and 0.96 for MgSiO_3_-DB30C10, it can be stated that this model does not accurately describe the Pd(II) adsorption process. Based on the pseudo-first-order kinetic model, the calculated adsorption capacities are also evaluated (q_e,calc_),with values differing very much from the experimental values of the adsorption capacities (q_e,exp_).

The results obtained using the pseudo-second-order kinetic model for modelling the experimental data, obtained based on the function t/q_t_ = f(t), using materials studied at the three temperatures, are represented in Figure 10. The calculated kinetic parameters associated with the pseudo-second-order kinetic model are presented in Table 2.

The values of the regression coefficient R^2^ being very close to the unity suggests that the pseudo-second order kinetic model describes very well the adsorption processes of Pd(II). The calculated value of adsorption capacity (q_e,calc_) is very close to the experimental values of the adsorption capacity (q_e,exp_), a fact that supports the validity of this model. This is based on the hypothesis that in the process of Pd(II) adsorption on the two materials, the determining stage is a chemical process that takes place through the formation of strong chemical bonds established by electrostatic attraction and ion exchange between them and the substrate [30,31].

**Figure 11:**
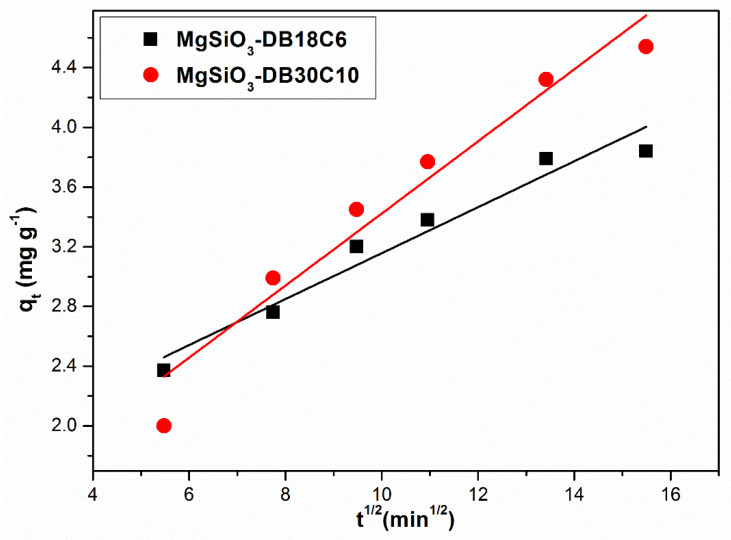
Intraparticle diffusion.

The possibility of intraparticle diffusion is further investigated. The particle agitation effect on intraparticle diffusion is insignificant since it increases the agitation degree, reducing the thickness of the boundary layer and thus only increases the external mass transfer coefficient. Figure 11 shows the intraparticle diffusion model specific to the Pd(II) adsorption process on MgSiO_3_-DB18C6 and MgSiO_3_-DB30C10 materials in the linearized form.

The values of the specific intraparticle diffusion model parameters are presented in Table 3. The value of the k_dif_ constants presented in Table 3 can be used to evaluate the influence of the studied parameters on the kinetics of the adsorption process: the higher the k_diff_ value, the lower the resistance encountered during the intraparticle diffusion process and therefore the faster the adsorption [32].

The adsorption process of Pd (II) on both materials can be modeled using the Weber and Morris model [33], with quite high accuracy, as it is confirmed by the values of the coefficients R^2^. Theoretically, the intraparticle diffusion specific equations indicate that the concentration dependence of the diffusion–adsorption process varies with the characteristics of the adsorption isotherm and with the amount of solute adsorbed at the equilibrium time [34]. Finally, the efficiency of the adsorption process may be a limiting factor for the kinetic effects.

The results analysis using the Weber and Morris model shows that: (1) the graphical representation is not characterized by a very good linearity nor does it pass through the origin, (2) in most cases the graphs show multilinearity and (3) constant C has no negative values. All of this indicates that intraparticle diffusion is not the only decisive step in the velocity of the adsorption process and the diffusion through the liquid film also plays an important role in controlling the adsorption kinetics.

#### 2.2.5. Adsorption Isotherm Study

**Figure 12:**
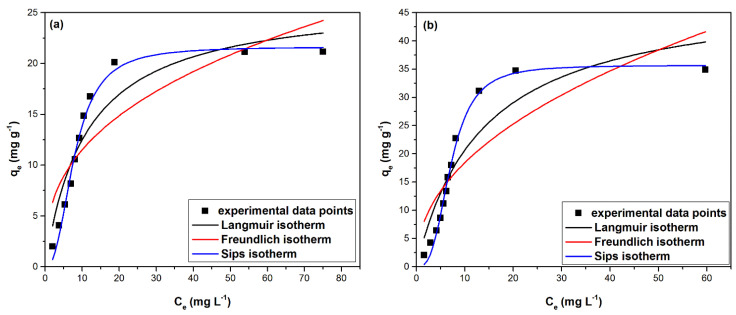
Langmuir, Freundlich and Sips isotherms: (**a**) MgSiO_3_-DB18C6, (**b**) MgSiO_3_-DB30C10.

For a better understanding of the adsorption process it is necessary to identify the adsorption mechanism, namely by describing how the solution interacts with the adsorbent material. This can be achieved by using equilibrium isotherms which illustrate the relationship between the amount of substance adsorbed per gram of adsorbent, at equilibrium (q_e_), and the concentration of metal ions remaining in the aqueous phase (C_e_) [35]. A clear image of the adsorption process of Pd(II) on the two materials is obtained by mathematically modeling the experimental data, using three adsorption isotherms namely Langmuir, Freundlich and Sips isotherms (Figure 12) based on the function q_e_ = f(C_e_). Table 4 presents the specific parameters of the three studied isotherms.

It can be observed that the increase of the initial concentration of the Pd(II) solution, leads to an increase of the adsorption capacity, reaching the maximum adsorption capacity, q_m,exp_, for equilibrium concentrations higher than 100 (mg L^−1^). The highest adsorption capacity is higher for MgSiO_3_-DB30C10, namely 34.7 (mg g^−1^), than for MgSiO_3_-DB18C6, namely ~20 (mg g^−1^).

Based on the data analysis from Table 4 it can be seen that at higher equilibrium concentrations, the adsorption capacity of materials with studied adsorbent properties tends to a constant value. This value represents the maximum adsorption capacity obtained experimentally (q_exp_) for the two studied materials.

Due to the fact that the values of the parameter 1/n_F_ are subunitary, it can be stated that the synthesized adsorbents have a high affinity for Pd(II), and also that the studied adsorption processes are favorable, based on the convex shapes of the adsorption isotherms. Considering that the values of the heterogeneity factor 1/n_F_ is 0.34 for MgSiO_3_-DB18C6 and 0.45 for MgSiO_3_-DB30C10, having a large deviation from the unit value, it can be said that the studied materials have heterogeneous surfaces. The data presented in Table 4 suggest that regardless of the extractant used for the functionalization of magnesium silicate, the correlation coefficient R^2^ has the lowest values for the Freundlich isotherm, which suggests that this model has the lowest accuracy in terms of describing the adsorption processes. The correlation coefficient for the Sips isotherm has the closest to the unity; therefore, this model can be considered to best describe the adsorption processes. It is also observed that q_m,exp_ ~ q_m,calc_ for both materials are studied.

The values of the separation factor range between 0 < R_S_ < 1 confirming that the isotherm has a convex shape and the adsorption of Pd(II) on both materials is favorable.

#### 2.2.6. Activation Energy and Thermodynamic Parameters

**Figure 13:**
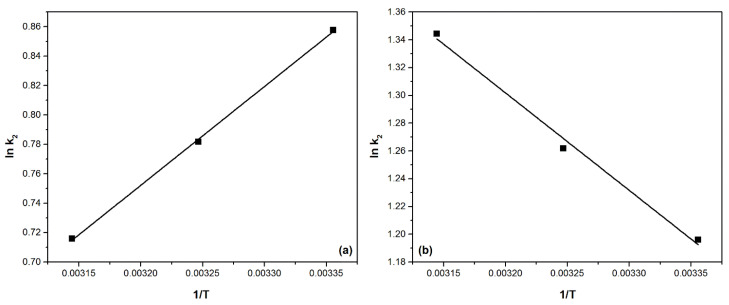
Activation energy for the Pd(II) adsorption process on the two studied materials: (**a**) MgSiO_3_-DB18C6, (**b**) MgSiO_3_-DB30C10.

The activation energy for adsorption processes is calculated based on the function ln (k_2_) = f(1/T) as presented in Figure 13 using the calculated values of the velocity constant (k_2_) obtained based on the pseudo-second-order kinetic model in Arrhenius’ equation.

Based on the linearized form of the pseudo-second-order kinetic model presented in the previous figures, the activation energy values associated with the Pd(II) adsorption processes on MgSiO_3_-DB18C6 and MgSiO_3_-DB30C10 are calculated (Table 5). The recovery process of Pd(II) is an adsorption process that can be consider a physical–chemical process [36].

**Figure 14:**
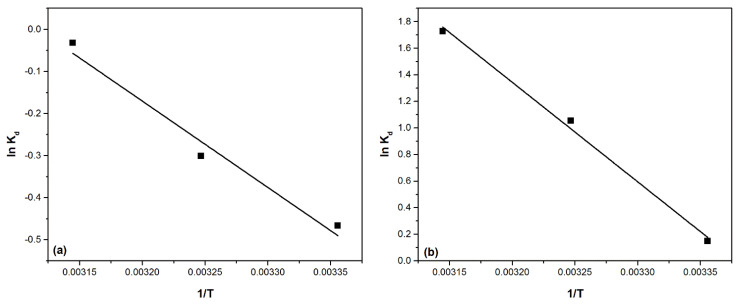
Thermodynamic studies for Pd(II) adsorption onto studied materials: (**a**) MgSiO_3_-DB18C6, (**b**) MgSiO_3_-DB30C10.

In order to establish the information regarding the energy changes associated with the adsorption process, thermodynamic studies are performed in the temperature range 298–318 K. Based on the obtained data from the thermodynamic studies, the spontaneous character of the adsorption processes can be specified. Thus, the variations of enthalpy (ΔH), Gibbs free energy (ΔG) and entropy (ΔS) are determined. From the linear representation of the dependence ln K_d_ = f(1/T) (Figure 14) the variation of entropy and, respectively, the variation of enthalpy are determined. Subsequently, the variation of Gibbs free energy is evaluated using the van’t Hoff equation.

Thermodynamic parameters calculated for Pd(II) adsorption on the two materials are presented in Table 6.

From the analysis of the data presented in Table 6 it can be seen that for all the materials studied, regardless of the working temperature, the variation of Gibbs free energy has negative values. This confirms that the Pd(II) adsorption process is a spontaneous process. It is also observed that, simultaneously with the increase of the working temperature, there is a decrease of the Gibbs free energy value, which confirms the positive effect of the temperature upon the adsorption process. Correlating the slight increase of the adsorption capacity simultaneously with the increase of the temperature and with the positive values of the enthalpy, it can be stated that the studied adsorption processes are endothermic. The positive values of the entropy (ΔS^0^) suggest that the studied adsorption processes show a higher disorder at the liquid/solid interface. However, the values of the entropy variation are relatively high, which suggests that there are major changes in the degree of disorder at the interface. It is also observed that the ΔS^0^ value for the adsorption of Pd(II) on the MgSiO_3_-DB30C10 material is much higher compared to MgSiO_3_-DB18C6 material, suggesting that the changes at the MgSiO_3_-DB30C10 interface are significant.

#### 2.2.7. Comparison of the Materials in the Study with the Literature Precedents

Table 7 presents previous literature data for different adsorbent materials used for Pd(II) recovery. The synthesized materials presented in this paper have higher adsorption capacities than many other materials presented in the specialized literature. The adsorbents mentioned in Table 7 cover a wide spectrum of materials from bio-polymers to nanomaterials.

#### 2.2.8. Desorption Studies

It is known that the use of materials having adsorbent properties depends not only on its adsorption capacity, but also on its ability to regenerate and then reuse. In order to be able to reuse an adsorbent material, it is necessary to be able to easily desorb the metal from its surface, obviously in a sufficiently large amount to make it cost-effective to reuse it. In this sense, the possibility of reusing MgSiO_3_-DB18C6 and MgSiO_3_-DB30C10 materials after Pd(II) desorption is also followed.

The desorption, conducted using HNO_3_ 5, 10 and 15%, proves to be optimal when using 10% HNO_3_, the highest amount of desorbed Pd(II) being 98%. Using a higher HNO_3_ concentration (15%) leads to a very small increase of the degree of desorption, 98.3%, suggesting that a concentration higher than 10% of HNO_3_ is not required.

#### 2.2.9. Adsorption Mechanism Prediction

The selective extraction of metal ions traces from unconventional sources such as wastewater is a long-term challenge, due to the large concentration difference between the target metal ions and the interfering ions in the matrix.

In this study we present two adsorbent materials obtained by the impregnation operation of MgSiO_3_ with two crown ethers of different sizes, DB18C6 and DB30C10. By crown ether grafting on the MgSiO_3_ surface, the possibility of selective complexation of the Pd(II) ion is increased. These new adsorbent materials have structures with large specific surfaces and pore volume, which favor the selective adsorption of Pd(II) from an aqueous solution. This grafting/functionalization takes place through hydrogen bridges created between O-H present on the surface of magnesium silicate and O from the crown ether (Stage I of the mechanism).

Crown ether grafted onto MgSiO_3_ offers high efficiency in the recovery of Pd(II) from aqueous solutions, forming the sandwich complexes known in the literature [27,44,45]. The selectivity of crown ethers depends on the compatibility between the size Pd(II) ions and the size of the crown cavity (number of O atoms of the crown) [27].

In the second stage of the mechanism, the chelation of the metal ion in the crown ether takes place.

Thus, the MgSiO_3_-DB30C10 material having a larger, almost double, number of O and C atoms from the crown ether compared to MgSiO_3_-DB18C6 allows two Pd(II) ions to occupy the crown cavity compared to only one Pd(II) ion for DB18C6.

**Figure 15:**
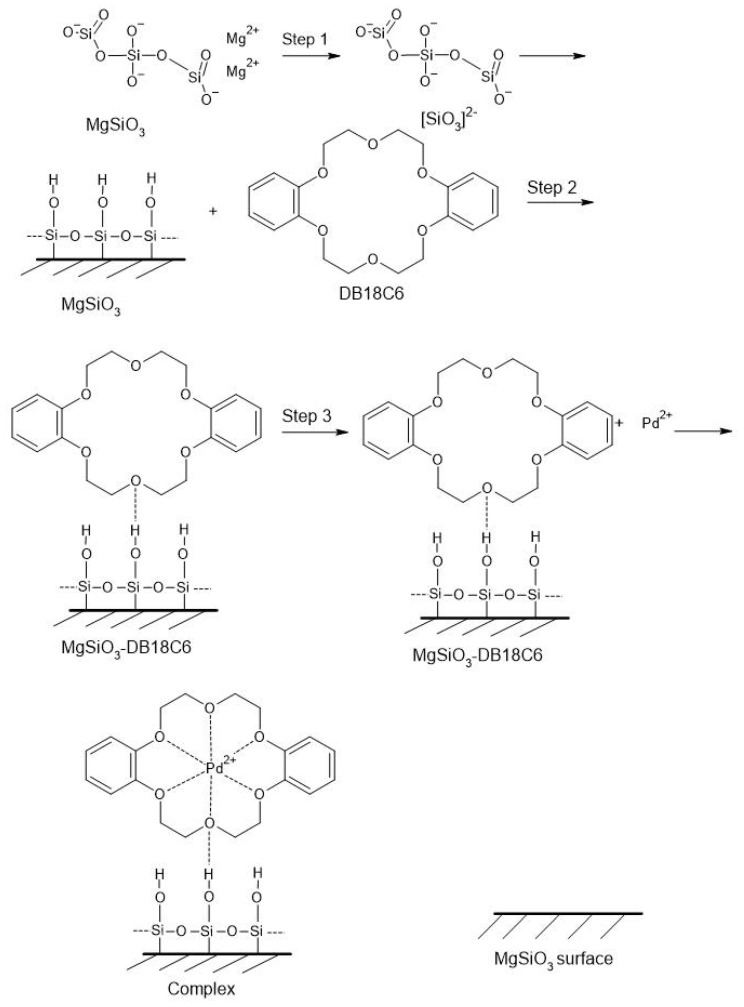
Mechanism for MgSiO_3_-DB18C6.

**Figure 16:**
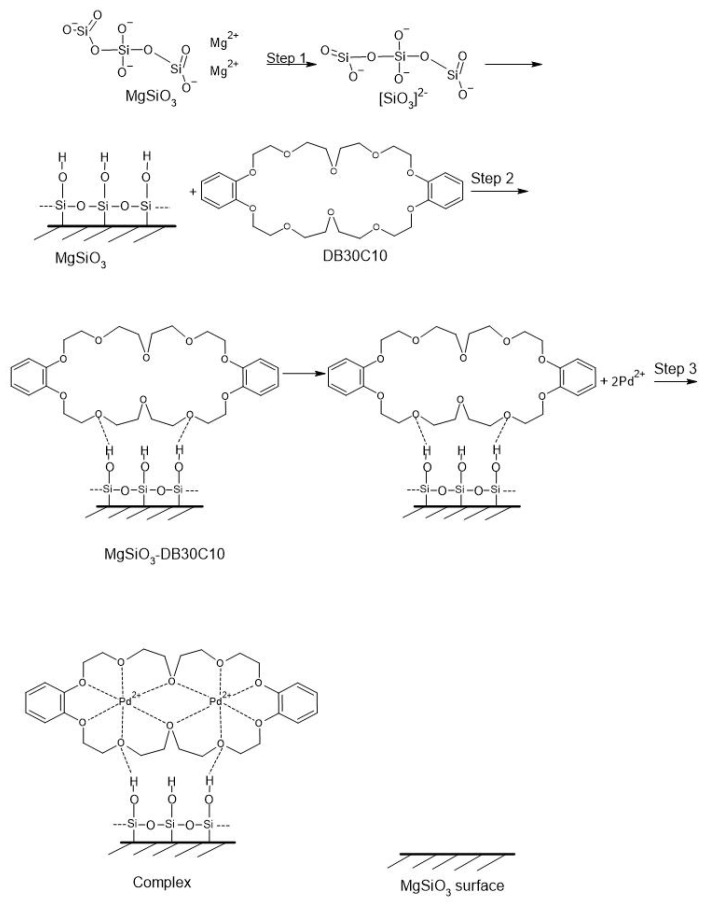
Mechanism for MgSiO_3_-DB30C10.

The proposed mechanisms of impregnation with crown ethers and retention of palladium ions are presented in the Figure 15 and Figure 16.

## 3. Materials and Methods

The raw materials for the new adsorbents’ synthesis are magnesium silicate, dibenzo-18-crown-6 and dibenzo-30-crown-10. The structure and some of the properties of these materials are briefly presented in Table 8.

The two adsorbent materials, MgSiO_3_-DB18C6, and MgSiO_3_-DB30C10, were obtained by functionalization using an MgSiO_3_: crown ether ratio of 10:1. For the synthesis, 0.1 g of each dibenzo-18-crown ether-6 acid extractor (Sigma-Aldrich/Merck, London, UK, purity 98%) and dibenzo-30-crown-10 acid (Sigma-Aldrich/Merck, purity 98%) were weighed and then dissolved in 25 mL nitrobenzene (99%, Carl Roth).

The functionalization was realized using the dissolved extractant and 1 g of MgSiO_3_ (60–100 mesh, Merck, Berlin, Germany), kept in contact for 24 h, then dried in an oven (POL-EKO SLW53, Poland) for 24 h at 323 K. The method used to functionalize the inorganic substrate was the SIR (solvent impregnated resin) dry method [46,47].

The obtained materials were characterized using scanning electron microscopy, SEM and X-ray energy dispersion (EDX), with an FEI Quanta FEG 250a X-ray energy dispersion spectrometer. The solid samples were placed on a self-adhesive carbon paper surface recommended by the SEM manufacturer. They were fixed on stabs in the device and SEM images were taken in a low vacuum in order to avoid the surface charges so that no electric discharges would appear.

A Fourier-transform infrared spectroscopy, FT-IR, analysis of the synthesized materials MgSiO_3_-DB18C6 and MgSiO_3_-DB30C10 was performed using a Bruker Platinum ATR-QL Diamond FT-IR spectrometer, in the range 4000–400 cm^−1^.

The specific surface area and the porosity of the obtained materials were measured using the BET (Brunauer–Emmett–Teller) method with a Nova 1200e Quantachrome apparatus.

The point of zero charge (pZc) was determined using the batch equilibrium technique [48,49]. For this study an amount of 0.1 g of MgSiO_3_-DB18C6 and MgSiO_3_-DB30C10 was mixed (water bath with thermostat and stirring type Julabo SW23, 200 rotations/minute and a temperature of 298 K) with 25 mL of the 0.01 M KCl solution whose pH was adjusted in the range 1–14 using NaOH or HNO_3_ solutions (concentration range 0.05 N to 2 N). The samples were filtered, and afterwards the resulted solution’s pH was determined using a Mettler Toledo pH meter, SevenCompact.

The adsorption studies were made on a 1000 (mg L^−1^) synthetic Pd(II) aqueous solution of Pd(NO_3_)_2_, (Certipur, Merck, Germany). The efficiency of the two adsorbent materials, MgSiO_3_-DB18C6, and MgSiO_3_-DB30C10 for Pd(II) recovery from aqueous solutions, the influence of specific parameters such as pH, contact time, temperature and initial concentration upon the adsorption capacity were studied.

The variation of the adsorbent adsorption capacity with the pH provides information about the effect of the acidity of the solution containing the metal ion on the surface of the adsorbent material. Thus, in this paper the pH ranges from 2 to 10, for an initial 25 mL solution Pd(II) concentration C_0_ = 20 (mg L^−1^), using 0.1 g adsorbent, a contact time of 1 h and a temperature of 298 K. The pH of the solution was measured using the Mettler Toledo pH meter, SevenCompact.

The influence of contact time and temperature on the adsorption capacity of MgSiO_3_-Db18C6 and MgSiO_3_-Db30C10 was determined using precisely 0.1 g of material immersed in 25 mL of Pd(II) solution of concentration C_0_ = 20 (mg L^−1^). The samples were stirred at different times (30, 60, 90, 120, 180 and 240 min) in a Julabo SW23 thermostatic bath and stirred (200 rpm) at different temperatures (298 K, 308 K and 318 K).

To study the effect of the initial Pd(II) concentration on the adsorption capacity of the two materials, Pd(II) solutions of different concentrations were prepared and then mixed with a fixed amount of 0.1 g adsorbent material using a contact time of 2 h at a temperature of 298 K and pH = 3 for a sample volume of 25 mL. Thus, for MgSiO_3_-DB18C6 the concentrations are: 10, 20, 30, 40, 50, 60, 70, 80, 100, 140 and 160 (mg L^−1^) and for MgSiO_3_-DB30C10 the concentrations are: 10, 20, 30, 40, 50, 60, 70, 80, 100, 140, 160 and 200 (mg L^−1^).

The residual metal ion concentration was measured using an atomic absorption spectrophotometer Varian, SpectrAA 280 FS.

A solid–liquid sorption system is usually evaluated by performing equilibrium tests and dynamic studies [50,51]. In the adsorption process, the dissolved palladium ions bind to the adsorbent through physical or physico–chemical interactions until equilibrium is reached. The adsorption capacity at equilibrium is calculated using the following equation:

q_e_ = (C_0_ − C_e_)∙V/m (1)

where:

- q_e_—equilibrium adsorption capacity (mg g^−1^),
- C_0_—the initial concentration of palladium in solution (mg L^−1^),
- C_e_—the equilibrium concentration of palladium in solution (mg L^−1^),
- V—palladium solution volume (L), m—adsorbent quantity (g).

Kinetic models are used to identify the type of adsorption mechanism for the studied system and the potential steps to control the velocity, including mass transport processes and chemical reactions [52]. The most commonly used are pseudo-first-order kinetic models (Lagergren model) [53] and pseudo-second-order models (Ho and McKay model) [30].

The mathematical equations characterizing the pseudo-first-order (Lagergren model) is:

ln (q_e_ − q_t_) = ln q_e_ − k_1_∙t (2)

where:

- q_e_—equilibrium adsorption capacity (mg g^−1^),
- q_t_—adsorption capacity at t time (mg g^−1^),
- k_1_—pseudo first-order constant (min^−1^), t—contact time (min).

The mathematical equations characterizing the pseudo-second-order (the Ho and McKay model) is:

t/q_t_ = 1/k_2_∙q_e_^2^ + t/q_e_ (3)

where:

- q_e_—equilibrium adsorption capacity (mg g^−1^),
- q_t_—adsorption capacity at t time (mg g^−1^),
- k_2_—pseudo second-order constant (g mg^−1^ min^−1^),
- t—contact time (min).

From the linear fit of the function ln (q_e_–q_t_) = f(t), the rate constant for the pseudo- first-order k_1_ and the adsorption capacity q_e,calc_ can be calculated. Similarly, from the linear fit of the function t/q_t_ = f(t) the rate constant for the pseudo- second-order k_2_ and the adsorption capacity q_e,calc_ can be determined.

Based on the kinetic parameters calculated for each model, it is possible to establish the model that describes exactly the adsorption process of Pd(II) on the synthetized adsorbents MgSiO_3_-DB18C6 and MgSiO_3_-DB30C10.

The activation energy E_a_ can be calculated using the Arrhenius equation and the kinetic rate constant of the model of the pseudo-second-order k_2_, which is specific for the Pd(II) adsorption process on the two materials. The equation is:

ln k_2_ = ln A − E_a_/RT (4)

where:

- k_2_—speed constant (g min^−1^ mg^−1^),
- A—Arrhenius constant (g min mg^−1^),
- E_a_—activation energy (kJ mol^−1^),
- T—absolute temperature (K),
- R—ideal gas constant (8.314 J mol^−1^ K^−1^).

The linear fit for equation ln k_2_ = f(1/T) can be used to calculate the activation energy for the Pd(II) adsorption process on the studied adsorbents MgSiO_3_-DB18C6 and MgSiO_3_-DB30C10.

The adsorption process on porous adsorbents goes through the following steps: (1) transporting the adsorbate from the solution to the liquid film surrounding the adsorbent; (2) transporting the adsorbate through the liquid film to the outer surface of the adsorbent (film diffusion); (3) transport of the adsorbate from the external surface of the adsorbent inside its pores (intraparticle diffusion); (4) retention of the adsorbate inside the pores by physical, chemical or ion exchange adsorption. Usually, steps (1) and (4) are very fast and cannot represent the decisive stages that affect the velocity of the adsorption process.

To distinguish whether film diffusion or intraparticle diffusion is the determinant stage, kinetic experimental data were processed according to the Weber and Morris model [33]:

q_t_ = k_diff_ ∙t^1/2^ + C (5)

where:

- q_t_—adsorption capacity at t time; k_diff_—intraparticle diffusion speed constant (mg g^−1^ min^−1/2^);
- C—a constant correlated with the thickness of the liquid film surrounding the adsorbent particles.

In order for the intraparticle diffusion to be the only determining stage for the adsorption velocity, it is necessary that the graph of the function q_t_ = f(t^1/2^) is very close to a line passing through the origin (C = 0). Otherwise, both intraparticle diffusion and film diffusion influence the adsorption kinetics. A negative value of C also indicates that film diffusion affects the adsorption kinetics.

The adsorption capacity of the experimental materials MgSiO_3_-DB18C6 and MgSiO_3_-DB30C10 was described using three models: Langmuir, based on the monolayer adsorption of solute, Freudlich which was originally developed for heterogeneous surfaces and Sips, a model that combines the two previous ones [54,55].

The Langmuir model is based on 3 hypotheses: (i) adsorption takes place only in a single layer; (ii) all surface voids are identical, housing a single metal ion; and (iii) the ability of a molecule to be adsorbed on a surface is independent of the occupation of adjacent sites [56].

The nonlinear form of the Langmuir isotherm is [57]:

q_e_ = q_L_∙K_L_∙C_e_/(1 + K_L_∙C_e_) (6)

where:

- q_e_—equilibrium adsorption capacity (mg g^−1^),
- C_e_—metal ion equilibrium concentration from solution (mg L^−1^),
- qL—Langmuir maximum adsorption capacity (mg g^−1^), K_L_–Langmuir constant.

The Freundlich isotherm is an empirical isotherm [58], and the equation widely used to explain the equilibrium of the adsorption process is:

q_e_ = K_F_∙C_e_^1/nF^ (7)

where:

- q_e_—equilibrium adsorption capacity (mg g^−1^),
- C_e_—metal ion equilibrium concentration from solution (mg g^−1^), K_F_ and
- n_F_—characteristic constants that can be associated with the relative adsorption capacity of the adsorbent or the adsorption intensity.

The Sips model, also called the Langmuir–Freundlich model, is characterized by the mathematical equation [59]:

q_e_ = q_S_∙K_S_∙C_e_^1/nS^/(1 + K_S_∙C_e_^1/nS^) (8)

where:

- q_S_—maximum adsorption capacity (mg g^−1^),
- K_S_—constant related to the adsorbent adsorption capacity,
- n_S_—heterogeneousness factor.

The mechanism that explains how the Pd(II) adsorption occurs on MgSiO_3_-DB18C6 and MgSiO_3_-DB30C10 materials, was established based on the Gibbs free energy value calculated using the Gibbs–Helmholtz equation [60]:

∆G^0^ = ∆H0 − T∙∆S^0^ (9)

where:

- ΔG^0^—Gibbs free energy standard variation (kJ mol^−1^),
- ΔH^0^—enthalpy standard variation (kJ mol^−1^),
- ΔS^0^—entropy standard variation (J mol^−1^ K^−1^),
- T—absolute temperature (K).

From the equation describing the linear fit of the function the graphical representation of ln K_d_ = f(1/T), one can calculate the standard entropy variation ΔS^0^ and the standard enthalpy variation ΔH^0^:

ln K_d_ = ∆S^0^/R − ∆H^0^/RT (10)

where:

- K_d_—equilibrium constant,
- ΔS^0^—entropy standard variation (J mol^−1^ K^−1^),
- ΔH^0^—enthalpy standard variation (kJ mol^−1^),
- T—absolute temperature (K),
- R—ideal gas constant 8.314 (J mol^−1^ K^−1^).

The equilibrium constant K_d_ is defined by the ratio between the adsorption capacity at equilibrium q_e_ and the equilibrium concentration C_e_.

K_d_ = q_e_/C_e_ (11)

In the desorption process, the Pd(II) ions bound on the adsorbent material were desorbed by mixing with 250 mL HNO_3_ solutions having different concentrations, namely 5%, 10% and 15%. The samples were stirred for 6 h at 300 rpm at room temperature. The material was then washed with distilled water and dried at room temperature. The efficiency of the desorption process was established, considering the Pd(II) amount desorbed using the following relation:

% Desorption ratio = C_d_∙100/C_e_ (12)

where:

- C_d_—desorption concentration of Pd(II).

## 4. Conclusions

In this study we report the synthesis of two new adsorbent materials using the SIR method starting from magnesium silicate and crown ethers (DB18C6 and DB30C10). These materials have distinct structures, presenting specific surfaces and different pore volumes influenced by the size of the crown ethers. It is one of the reasons why these materials have good efficiencies in terms of the recovery of Pd(II) from aqueous solutions by adsorption. Adsorption occurs spontaneously after ~120 min, reaching equilibrium. Correlating the slight increase of the adsorption capacity simultaneously with the increase in the temperature and with the positive values of the enthalpy, it can be stated that the studied adsorption processes are endothermic. Positive values of entropy suggest that the studied adsorption processes show a greater disorder at the liquid/solid interface. However, the values of the entropy variation are relatively high, suggesting that there are major changes in the degree of disorder at the interface. A mechanism of the adsorption process was predicted, through which it was established that the MgSiO_3_-DB30C10 material being of higher crown ether allows the complexation of two molecules of Pd(II) in a molecule of DB30C10, confirmed by the higher adsorption value (~34.7 mg g^−1^) compared to MgSiO_3_-DB18C6 (~20 mg g^−1^), where the crown ether DB18C6 allows only one molecule of Pd(II) to be complexed.

## Figures and Tables

**Table 1:** Textural parameters calculated from N_2_ adsorption isotherms.

Sample	BET Surface Area (m^2^ g^−1^)	Pore Width (nm)	Total Pore Volume (cm^3^ g^−1^)	Fractal Dimension
MgSiO_3_	205	3.87	0.33	66.6
MgSiO_3_-DB18C6	75	1.69	0.03	2.65
MgSiO_3_-DB30C10	215	2.89	0.48	69.5

**Table 2:** Kinetic parameters for Pd(II) adsorption onto adsorbent materials.

Temperature (K)	q_e,exp_ (mg g^−1^)	Pseudo-First Order Kinetic Model			Pseudo-Second Order Kinetic Model		
		q_e,calc_ (mg g^−1^)	k_1_ (min^−1^)	R^2^	q_e,calc_ (mg g^−1^)	k_2_ (g mg^−1^ min^−1^)	R^2^
MgSiO_3_-DB18C6							
298	3.58	1.60	0.0059	0.9147	3.72	2.3579	0.9974
308	3.99	2.02	0.0065	0.9305	3.84	2.1850	0.9966
318	4.20	2.29	0.0075	0.9501	3.99	2.0462	0.9974
MgSiO_3_-DB30C10							
298	4.11	2.06	0.0075	0.8656	4.34	2.1496	0.9917
308	4.59	2.51	0.0091	0.8456	4.82	3.1390	0.9974
318	4.78	2.65	0.0975	0.9627	5.10	4.4606	0.9966

**Table 3:** The intraparticle diffusion model (IPD) parameters for Pd(II) adsorption onto adsorbent materials.

Adsorbent Material	Intraparticle Diffusion Model (IPD)		
	K_diff_ (mg g^−1^ min ^−1/2^)	C	R^2^
MgSiO_3_-DB18C6	1.62	0.15	0.9479
MgSiO_3_-DB30C10	1.00	0.24	0.8969

**Table 4:** Langmuir, Freundlich and Sips isotherm parameters for Pd(II) ions’ adsorption onto adsorbent materials.

Material	q_m,exp_ (mg g^−1^)	Langmuir Isotherm			Freundlich Isotherm			Sips Isotherm			
		q_L_ (mg g^−1^)	K_L_	R^2^	K_F_ (mg g^−1^)	1/n_F_	R^2^	K_S_	q_S_ (mg g^−1^)	1/n_S_	R^2^
MgSiO_3_-DB18C6	21.12	26.41	0.089	0.8800	4.91	0.36	0.7206	0.0026	21.65	0.06	0.9980
MgSiO_3_-DB30C10	34.80	48.9	0.073	0.8421	6.45	0.45	0.6885	0.0013	35.68	0.02	0.9910

**Table 5:** Activation energy and correlation coefficient for the Pd(II) adsorption process.

Adsorbent Materials	Activation Energy (kJ mol^−1^)	R^2^
MgSiO_3_-DB18C6	55.8	0.9994
MgSiO_3_-DB30C10	58.3	0.9999

**Table 6:** Thermodynamic parameters for Pd(II) adsorption onto adsorbent.

Temperature (K)	MgSiO_3_-DB18C6			MgSiO_3_-DB30C10		
	ΔG^0^ (kJ mol^−1^)	ΔH^0^ (kJ mol^−1^)	ΔS^0^ (kJ mol^−1^ K^−1^)	ΔG^0^ (kJ mol^−1^)	ΔH^0^ (kJ mol^−1^)	ΔS^0^ (kJ mol^−1^ K^−1^)
298	−15.82	17.05	53.16	−62.67	62.3	210.5
308	−16.36			−64.77		
318	−16.88			−66.88		

**Table 7:** Comparison of the materials in the study with the literature precedents.

Material	Adsorption Capacity (mg g^−1^)	Reference
Polyamine functionalized polystyrene-based beads	0.2	[37]
Cross-linked carboxymethylchitin and carboxymethylchitosan hydrogels	2.68	[38]
Polyamine functionalized polystyrene-based beads and nanofibers	4.3	[37]
2-Mercaptobenzothiazole impregnated cellulose	5	[39]
Chitosan	5.88	[40]
Nanofire de α-MnO_2_ α-MnO_2_ nanorods	7.9	[41]
Amberlite XAD-16 functionalized with 2-acteyl pyridine	8	[42]
Alumina loaded with 5-bromo-2-pyridylazo-5-diethylaminophenol	11	[43]
Polyethylenimine (PEI) onto alumina	13	[44]
2-Mercaptobenzimidazole impregnated chitosan	19.26	[40]
MgSiO_3_-DB18C6	20	This paper
MgSiO_3_-DB30C10	34.7	This paper

**Table 8:** Materials’ structure and properties.

Materials	Structure	Properties
Magnesium silicate (MgSiO_3_)	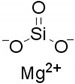	Density: 3.21 (g/cm^−3^) Melting point: 191 °C Particle size: 0.15–0.25 mm
Dibenzo 18-crown-6 (DB18C6)	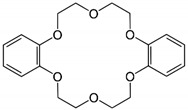	Melting point: 162–164 °C Boiling point: 380–384 °C Density: 1.1801 (g/cm^−3^) Solubility: 0.007 (g/L^−1^) Form: fluffy powder
Dibenzo 30-crown-10 (DB 30C10)	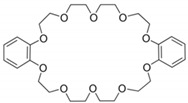	Melting point: 106–108 °C Boiling point: 573.63 °C Density: 1.1391 (g/cm^−3^) Form: white crystalline powder

## Data Availability

All the experimental data are presented, in the form of a table and/or figure, in the article.
